# The Role of Lipid-Lowering Treatment in the Secondary Prevention of Ischemic Stroke

**DOI:** 10.3390/diseases10010003

**Published:** 2021-12-27

**Authors:** Alexandra Tsankof, Konstantinos Tziomalos

**Affiliations:** First Propedeutic Department of Internal Medicine, Medical School, Aristotle University of Thessaloniki, AHEPA Hospital, 54636 Thessaloniki, Greece; a.tsankof@gmail.com

**Keywords:** ischemic stroke, secondary prevention, lipids, statins, ezetimibe, proprotein convertase subtilisin/kexin type 9 inhibitors, icosapent ethyl

## Abstract

Dyslipidemia is a major modifiable risk factor for ischemic stroke. Treatment with statins reduces the incidence of recurrent ischemic stroke and also reduces coronary events in patients with a history of ischemic stroke. Therefore, statins represent an important component of secondary prevention of ischemic stroke. In patients who do not achieve low-density lipoprotein cholesterol (LDL-C) targets despite treatment with the maximal tolerated dose of a potent statin, ezetimibe should be added to their lipid-lowering treatment and also appears to reduce the risk of cardiovascular events. Selected patients who do not achieve LDL-C targets despite statin/ezetimibe combination are candidates for receiving proprotein convertase subtilisin/kexin type 9 (PCSK9) inhibitors. Finally, it appears that adding icosapent ethyl might also reduce cardiovascular morbidity in patients who have achieved LDL-C targets but have persistently elevated triglyceride levels.

## 1. Introduction

Ischemic stroke represents the fourth leading cause of death and the first cause of long-term disability in high-income countries [1,2]. High low-density lipoprotein cholesterol (LDL-C) levels are a major modifiable risk factor for ischemic stroke [3]. High levels of triglycerides and low levels of high-density lipoprotein cholesterol (HDL-C) are also related to increased risk for ischemic stroke [4,5]. Moreover, in the INTERSTROKE study (*n* = 13,447 patients with stroke and 13,472 controls from 32 countries), 26.8% of strokes were due to an increased apoB/ApoA1 ratio [6]. Elevated total cholesterol levels are also a risk factor for carotid stenosis, a major cause of ischemic stroke [7]. In addition, randomized trials in patients with ischemic stroke showed a reduction in cardiovascular morbidity after treatment with a statin compared with placebo [8,9]. Therefore, the administration of statins represents an integral part of the secondary prevention of ischemic strokes according tocurrent guidelines [10,11]. However, the role of lipid-lowering treatment in some subtypes of ischemic stroke (e.g., cardioembolic and lacunar), as well as of other lipid-lowering agents except statins, is less clear. Additional confusion is created by the association between low LDL-C levels and increased risk for hemorrhagic stroke [1], as well asby reports that aggressive statin treatment might increase the risk of hemorrhagic stroke in patients with a history of an ischemic stroke [8].

The present review summarizes the current evidence regarding the role of lipid-lowering treatment in the secondary prevention of ischemic stroke.

## 2. The Role of Statins in Patients with Ischemic Stroke

Two large, randomized, placebo-controlled studies assessed the effects of statins on cardiovascular morbidity and mortality in patients with ischemic stroke (Table 1) [8,9]. In a subgroup analysis of the Heart Protection Study, 3280 patients with a history of non-hemorrhagic, non-disabling stroke (64%), transient ischemic attack (TIA, 26%), or carotid endarterectomy or stenting (10%), within 6 months before enrollment in the study, were randomized to receive simvastatin 40 mg or placebo [8]. After a mean follow-up of 4.8 years, simvastatin reduced the risk of major cardiovascular events (non-fatal myocardial infarction (MI) or coronary heart disease (CHD) death, stroke of any type, or any revascularisation procedure) by 20% compared with placebo (*p* = 0.001) [8]. Treatment with simvastatin did not reduce the incidence of ischemic stroke [8]. Rats of hemorrhagic stroke also did not differ between patients who received simvastatin and placebo [8].

The second large randomized trial that evaluate the effect of statins on cardiovascular events in patients with a prior stroke is the Stroke Prevention by Aggressive Reduction in Cholesterol Levels (SPARCL) [9]. In SPARCL, 4731 patients with a history of ischemic stroke (67%), TIA (31%), or hemorrhagic stroke (2%) within1–6 months prior to enrollment in the study, and without a history of CHD, were randomized to receive atorvastatin 80 mg daily or placebo [9]. After a mean follow-up of 4.9 years, patients who received atorvastatin had a 16% lower risk of developing a fatal or non-fatal stroke (*p* = 0.03) [9]. This decrease was due to the significant reduction in the risk of ischemic stroke by 22%, while there was an increase in the incidence of hemorrhagic stroke by 66% [9]. However, as ischemic strokes were considerably more common than hemorrhagic strokes, the absolute reduction in the number of ischemic strokes with atorvastatin (56 less events) was greater than the absolute increase in the number of hemorrhagic strokes (22 additional events) [9]. Moreover, patients receiving atorvastatin showed a 43% reduction in the risk of fatal stroke [9]. At the same time, the administration of atorvastatin reduced the risk of major cardiovascular events (non-fatal MI or stroke and cardiovascular mortality) by 20% (*p* = 0.002) [9]. In addition, in a meta-analysis of 31 randomized, controlled trials (*n* = 182,803), statins did not increase the risk of hemorrhagic stroke, and the latter was not associated with the degree of LDL-C-lowering or achieved LDL-C levels [13]. In another meta-analysis of 19 observational studies, there was also no effect of statin treatment on the incidence of hemorrhagic stroke [14].

The significant benefits of statins in reducing cardiovascular events in patients with a history of stroke are also confirmed by a meta-analysis of 26 randomized controlled trials comparing statins with placebo that included 169,138 patients [15]. In this meta-analysis, a decrease of LDL-C levels by 39 mg/dL with statins in patients with cardiovascular disease (CVD) but without CHD (i.e., mainly with stroke) reduced cardiovascular events by 19% [15]. The benefit was similar to the benefit of statin use in patients with CHD, who experienced a 21% reduction in cardiovascular events for a decrease in LDL-C levels by 39 mg/dl [15]. Accordingly, current guidelines of the European Society of Cardiology and the American Heart Association recommend high-intensity statin treatment in all patients with a history of ischemic stroke [10,11].

The non-significant reduction in the incidence of stroke in the Heart Protection Study might have been due to the smaller number of patients compared with SPARCL, resulting in a lack of statistical power [8,9]. Another possible explanation is that patients in the Heart Protection Study had suffered a stroke a mean of 4.3 years prior to enrolling in the study, while in the SPARCL study within one semester [8,9]. It is known that the risk of recurrent stroke is higher during the first year after the event [16,17], which also indicates the importance of initiating statin administration immediately after an ischemic stroke. At the same time, starting treatment with statins during hospitalization also improves adherence [18]. Another explanation is that the reduction in LDL-C levels was smaller in Heart Protection Study (39 vs. 56 mg/dL in SPARCL) [8,9]. However, despite the non-significant reduction of the risk of ischemic stroke in the Heart Protection Study, it should be noted that in both the latter study and in SPARCL, statin administration reduced the incidence of CHD events [8,9]. In a network meta-analysis, there was no significant difference between different statins in reducing the risk of stroke and higher doses were more protective against stroke [19].

Regarding LDL-C targets in patients with a history of ischemic stroke or TIA, a post-hoc analysis of SPARCL showed that patients who achieved LDL-C levels < 70 mg/dL had 34% lower risk of developing ischemic stroke and 42% lower risk of experiencing a CHD event, without an increase in the risk of hemorrhagic stroke, compared with patients who achieved LDL levels cholesterol > 100 mg/dL [20]. On the other hand, patients who achieved LDL-C levels 70–100 mg/dL did not show a significant reduction in cardiovascular events compared with patients who achieved LDL-C levels > 100 mg/dL [20]. Accordingly, current European and U.S. guidelines recommend reducing LDL-C levels to <55 and <70 mg/dL in patients with ischemic stroke, respectively [10,11].

Another important question regarding the administration of statins in patients with ischemic stroke is whether the benefit is the same in all subtypes of ischemic stroke. In a posthoc analysis of the SPARCL study, the reduction of cerebrovascular and major cardiovascular events was similar in patients with ischemic stroke due to vascular disease (atherothrombotic), in patients with ischemic stroke due to small vesseldisease (lacunar), and in patients with TIA [21]. It should be noted that 21% of patients in SPARCL had a history of ischemic stroke due to unknown etiology, and that these patients benefited equally from statin administration compared with patients with ischemic stroke due to large vessel disease [21]. On the other hand, the presence of atrial fibrillation was an exclusion criterion in SPARCL [9]. Moreover, in the HPS study, the effects of simvastatin on cardiovascular events in patients with different subtypes of ischemic stroke (i.e., atherothrombotic, lacunar, and cardioembolic) were not evaluated [8]. However, recent observational studies suggest that statins reduce cardiovascular events and all-cause mortality in patients with thromboembolic stroke (e.g., in the presence of atrial fibrillation) and that this reduction is similar to that in patients with non-embolic ischemic stroke [22,23]. Moreover, in patients with acute ischemic stroke and atrial fibrillation, the benefits of statins apply to all subgroups, including older patients, those with low cholesterol levels, patients receiving anticoagulants, and patients without clinical atherosclerotic cardiovascular disease, and are more prominent in patients who receive high-intensity statins than in those who are treated with low- or moderate-intensity statins [24].

## 3. Other Lipid-Lowering Agents in Patients with Ischemic Stroke

According to current European and U.S. guidelines, in patients who do not reach LDL-C targets despite treatment with the maximal tolerated dose of a potent statin (i.e., atorvastatin 40–80 mg or rosuvastatin 20–40 mg), ezetimibe should be added [10,11]. However, there are limited data regarding the effects of ezetimibe on cardiovascular events in patients with ischemic stroke. Indeed, the only study that evaluated whether the combination of ezetimibe and statins reduces cardiovascular events more than statin monotherapy is the Improved Reduction study Outcomes: Vytorin Efficacy International Trial (IMPROVE-IT), in which 18,144 patients with a recent hospitalization for an acute coronary syndrome (ACS) were randomized to receive simvastatin 40 mg combined with either ezetimibe or placebo [25]. In IMPROVE-IT, ezetimibe reduced major cardiovascular events by 6.4% compared with placebo (*p* = 0.016) [25]. The IMPROVE-IT study included 682 patients with a history of ischemic stroke, and in this subgroup, the reduction of cardiovascular events with ezetimibe was similar with the reduction in patients without a history of ischemic stroke [25].

In patients with a history of ischemic stroke who do not achieve LDL-C targets despite treatment with the maximal tolerated dose of a potent statin and ezetimibe, adding a proprotein convertase subtilisin/kexin type 9 (PCSK9) inhibitor should be considered according to current European and U.S. guidelines [10,11]. In the Further Cardiovascular Outcomes Research with PCSK9 Inhibition in Subjects with Elevated Risk (FOURIER) trial, 27,564 patients with established CVD with LDL-C levels > 70 mg/dL despite treatment with a statin were randomized to receive evolocumab 140 mg every 2 weeks or 420 mg every 4 weeks or placebo [12]. In the total study population, during a median follow-up of 2.2 years, evolocumab reduced the risk of ischemic stroke by 25% (*p* = 0.005) and did not affect the incidence of hemorrhagic stroke [12]. In the subgroup of patients with prior ischemic stroke (*n* = 5337), evolocumab yielded comparable reductions, even though effects were non-significant (hazard ratio 0.92, 95% confidence interval 0.68–1.25, *p* for interaction 0.09 compared with patients without a prior ischemic stroke) (Table 1) [12]. In the Evaluation of Cardiovascular Outcomes After an Acute Coronary Syndrome During Treatment With Alirocumab (ODYSSEY OUTCOMES) trial, 18,924 patients with had been hospitalized for ACS 1–12 months before enrollment and had LDL-C levels > 70 mg/dL despite treatment with atorvastatin 40–80 mg or rosuvastatin 20–40 mg were randomized to receive alirocumab 75–150 mg every 2 weeks or placebo [26]. In the total study population, during a median follow-up of 2.8 years, evolocumab reduced the risk of ischemic stroke by 27% without affecting the incidence of hemorrhagic stroke [26]. The effect of alirocumab on stroke was similar among patients with a history of previous cerebrovascular disease (*n* = 944) and among those without a history of cerebrovascular disease (*p* for interaction = 0.37) [26].

After LDL-C targets are achieved, if fasting triglycerides are 135–499 mg/dL, adding icosapent ethyl 2 g twice daily is recommended [10,11]. In the Japan EPA Lipid Intervention Study (JELIS), 18,645 Japanese patients with hypercholesterolemia were randomized to receivea statin combined with eicosapentaenoic acid (EPA) 1800 mg daily or statin only [27]. In the total trial population, EPA reduced major coronary events (death from CHD, fatal and non-fatal MI, unstable angina, coronary revascularization) by 19% [27]. In a sub-analysis of the JELIS study, the administration of EPA in 942 patients who had a history of prior stroke reduced the risk of stroke by 20% [28]. More recently, in the Reduction of Cardiovascular Events with Icosapent Ethyl-Intervention Trial (REDUCE-IT), 8179 patients with established CVD or diabetes with additional risk factors who were on statin treatment and had fasting triglyceride levels 135 to 499 mg/dL and LDL-C levels 41–100 mg/dL were randomly assigned to receive 2 g of icosapent ethyl twice daily or placebo [29]. After a median follow-up of 4.8 years, icosapent ethyl reduced the risk of any stroke by 28% [29]. Subgroup analyses of the effects of icosapent ethyl on the risk of recurrent stroke in have not been reported yet [29].

Finally, European guidelines mention that fibrates may be considered in combination with statins in patients with a history of ischemic stroke who are at LDL-C goals but have triglyceride levels > 200 mg/dL [10]. The only study that assessed the effect of fibrate–statin combination on cardiovascular events compared to statin monotherapy is the Action to Control Cardiovascular Risk in Diabetes (ACCORD) study, in which 5518 patients with diabetes who were receiving treatment with simvastatin were randomized to receive fenofibrate or placebo [30]. In the total study population, fenofibrate did not reduce the incidence of cardiovascular events [24]. However, in the subgroup of patients (*n* = 941) with triglycerides ≥ 204 mg/dL and HDL-C levels ≤ 34 mg/dL, the combination of simvastatin and fenofibrate reduced cardiovascular events by 31% [30]. The effect of simvastatin-fenofibrate combination in patients with a history of ischemic stroke included in the ACCORD study has not been reported yet [30].

## 4. Conclusions

**Figure 1:**
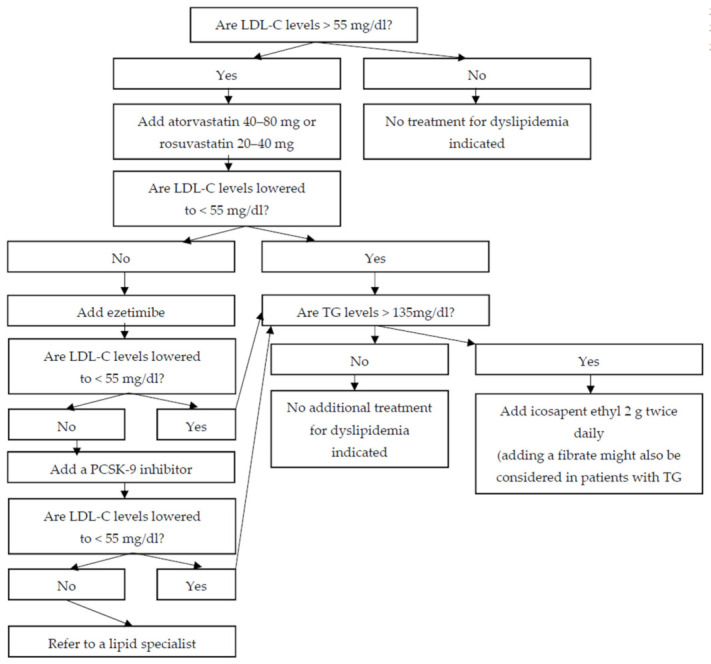
A proposed algorithm for the management of dyslipidemia in patients with a history of ischemic stroke (LDL-C: low-density lipoprotein cholesterol; TG: triglycerides; PCSK-9: proprotein convertase subtilisin/kexin type 9).

In patients with ischemic stroke, regardless of the underlying pathogenetic mechanism (i.e., atherothrombotic, embolic, or lacunar), administration of a potent statin to lower LDL-C levels < 55 mg/dL reduces the risk of both recurrent stroke and CHD. In patients who do not achieve LDL-C goals despite administration of the maximal tolerated dose of a potent statin, combination therapy with ezetimibe, and, if needed, a PCSK9 inhibitor, should be considered. More limited data suggest that icosapent ethyl might also be useful in patients with a history of ischemic stroke who have achieved LDL-C goals but have elevated triglyceride levels (Figure 1).

## Figures and Tables

**Table 1:** Major multicentre, randomized, double-blind, placebo-controlled trials that evaluated the effects of lipid-lowering agents on the secondary prevention of ischemic stroke (MI: myocardial infarction; SPARCL: Stroke Prevention by Aggressive Reduction in Cholesterol Levels; FOURIER: Further Cardiovascular Outcomes Research with PCSK9 Inhibition in Subjects with Elevated Risk).

Study	*n*	Active Treatment	Follow-Up (Years)	Major Outcomes
Heart Protection Study [8]	3280	Simvastatin 40 mg/day	4.8	Simvastatin reduced the risk of major cardiovascular events (non-fatal MI or coronary heart disease death, stroke of any type, or any revascularisation procedure) by 20% compared with placebo. Simvastatin did not affect the incidence of ischemic or hemorrhagic stroke.
SPARCL [9]	4731	Atorvastatin 80 mg/day	4.9	Patients who received atorvastatin had a 16% lower risk of fatal or non-fatal stroke, a 22% lower risk of ischemic stroke, a 43% lower risk of fatal stroke, a 20% lower risk of major cardiovascular events (non-fatal MI or stroke and cardiovascular mortality) but a 66% higher risk of hemorrhagic stroke.
FOURIER [12]	27,564	Evolocumab 140 mg every 2 weeks or 420 mg every 4 weeks	2.2	In the total study population, evolocumab reduced the risk of ischemic stroke by 25% and did not affect the incidence of hemorrhagic stroke. In the subgroup of patients with prior ischemic stroke (*n* = 5337), evolocumab yielded comparable reductions, even though effects were non-significant (hazard ratio 0.92, 95% confidence interval 0.68–1.25, *p* for interaction 0.09 compared with patients without a prior ischemic stroke).

## Data Availability

Not applicable.

## References

[1] Virani S.S., Alonso A., Aparicio H.J., Benjamin E.J., Bittencourt M.S., Callaway C.W., Carson A.P., Chamberlain A.M., Cheng S., Delling F.N. (2021). American Heart Association Council on Epidemiology and Prevention Statistics Committee and Stroke Statistics Subcommittee. Heart Disease and Stroke Statistics-2021 Update: A Report from the American Heart Association. Circulation.

[2] GBD 2019 Diseases and Injuries Collaborators (2020). Global burden of 369 diseases and injuries in 204 countries and territories, 1990–2019: A systematic analysis for the Global Burden of Disease Study 2019. Lancet.

[3] Iso H., Jacobs D.R., Wentworth D., Neaton J.D., Cohen J.D. (1989). Serum cholesterol levels and six-year mortality from stroke in 350,977 men screened for the multiple risk factor intervention trial. N. Engl. J. Med..

[4] Amarenco P., Labreuche J., Touboul P.J. (2008). High-density lipoprotein-cholesterol and risk of stroke and carotid atherosclerosis: A systematic review. Atherosclerosis.

[5] Labreuche J., Touboul P.J., Amarenco P. (2009). Plasma triglyceride levels and risk of stroke and carotid atherosclerosis: A systematic review of the epidemiological studies. Atherosclerosis.

[6] O’Donnell M.J., Chin S.L., Rangarajan S., Xavier D., Liu L., Zhang H., Rao-Melacini P., Zhang X., Pais P., Agapay S. (2016). Global and regional effects of potentially modifiable risk factors associated with acute stroke in 32 countries (INTERSTROKE): A case-control study. Lancet.

[7] Wilson P.W., Hoeg J.M., D’Agostino R.B., Silbershatz H., Belanger A.M., Poehlmann H., O’Leary D., Wolf P.A. (1997). Cumulative effects of high cholesterol levels, high blood pressure, and cigarette smoking on carotid stenosis. N. Engl. J. Med..

[8] Collins R., Armitage J., Parish S., Sleight P., Peto R., Heart Protection Study Collaborative Group (2004). Effects of cholesterol-lowering with simvastatin on stroke and other major vascular events in 20536 people with cerebrovascular disease or other high-risk conditions. Lancet.

[9] Amarenco P., Bogousslavsky J., Callahan A., Goldstein L.B., Hennerici M., Rudolph A.E., Sillesen H., Simunovic L., Szarek M., Welch K.M. (2006). High-dose atorvastatin after stroke or transient ischemic attack. N. Engl. J. Med..

[10] Authors/Task Force Members, ESC Committee for Practice Guidelines (CPG), ESC National Cardiac Societies (2019). 2019 ESC/EAS guidelines for the management of dyslipidaemias: Lipid modification to reduce cardiovascular risk. Atherosclerosis.

[11] Kleindorfer D.O., Towfighi A., Chaturvedi S., Cockroft K.M., Gutierrez J., Lombardi-Hill D., Kamel H., Kernan W.N., Kittner S.J., Leira E.C. (2021). 2021 Guideline for the Prevention of Stroke in Patients with Stroke and Transient Ischemic Attack: A Guideline from the American Heart Association/American Stroke Association. Stroke.

[12] McKinney J.S., Kostis W.J. (2012). Statin therapy and the risk of intracerebral hemorrhage: A meta-analysis of 31 randomized controlled trials. Stroke.

[13] Hackam D.G., Woodward M., Newby L.K., Bhatt D.L., Shao M., Smith E.E., Donner A., Mamdani M., Douketis J.D., Arima H. (2011). Statins and intracerebral hemorrhage: Collaborative systematic review and meta-analysis. Circulation.

[14] Baigent C., Blackwell L., Emberson J., Holland L.E., Reith C., Bhala N., Peto R., Barnes E.H., Keech A., Cholesterol Treatment Trialists’ (CTT) Collaboration (2010). Efficacy and safety of more intensive lowering of LDL cholesterol: A meta-analysis of data from 170,000 participants in 26 randomised trials. Lancet.

[15] Vickrey B.G., Rector T.S., Wickstrom S.L., Guzy P.M., Sloss E.M., Gorelick P.B., Garber S., McCaffrey D.F., Dake M.D., Levin R.A. (2002). Occurrence of secondary ischemic events among persons with atherosclerotic vascular disease. Stroke.

[16] Hardie K., Hankey G.J., Jamrozik K., Broadhurst R.J., Anderson C. (2004). Ten-year risk of first recurrent stroke and disability after first-ever stroke in the Perth Community Stroke Study. Stroke.

[17] Ovbiagele B., Saver J.L., Fredieu A., Suzuki S., Selco S., Rajajee V., McNair N., Razinia T., Kidwell C.S. (2004). In-hospital initiation of secondary stroke prevention therapies yields high rates of adherence at follow-up. Stroke.

[18] Tramacere I., Boncoraglio G.B., Banzi R., Del Giovane C., Kwag K.H., Squizzato A., Moja L. (2019). Comparison of statins for secondary prevention in patients with ischemic stroke or transient ischemic attack: A systematic review and network meta-analysis. BMC Med..

[19] Amarenco P., Goldstein L.B., Szarek M., Sillesen H., Rudolph A.E., Callahan A., Hennerici M., Simunovic L., Zivin J.A., Welch K.M. (2007). Effects of intense low-density lipoprotein cholesterol reduction in patients with stroke or transient ischemic attack: The Stroke Prevention by Aggressive Reduction in Cholesterol Levels (SPARCL) trial. Stroke.

[20] Amarenco P., Benavente O., Goldstein L.B., Callahan A., Sillesen H., Hennerici M.G., Gilbert S., Rudolph A.E., Simunovic L., Zivin J.A. (2009). Results of the Stroke Prevention by Aggressive Reduction in Cholesterol Levels (SPARCL) trial by stroke subtypes. Stroke.

[21] Ntaios G., Papavasileiou V., Makaritsis K., Milionis H., Manios E., Michel P., Lip G.Y., Vemmos K. (2014). Statin treatment is associated with improved prognosis in patients with AF-related stroke. Int. J. Cardiol..

[22] Choi J.Y., Seo W.K., Kang S.H., Jung J.M., Cho K.H., Yu S., Oh K. (2014). Statins improve survival in patients with cardioembolic stroke. Stroke.

[23] Choi K.H., Seo W.K., Park M.S., Kim J.T., Chung J.W., Bang O.Y., Kim G.M., Song T.J., Kim B.J., Heo S.H. (2019). Effect of Statin Therapy on Outcomes of Patients with Acute Ischemic Stroke and Atrial Fibrillation. J. Am. Heart Assoc..

[24] Cannon C.P., Blazing M.A., Giugliano R.P., McCagg A., White J.A., Theroux P., Darius H., Lewis B.S., Ophuis T.O., Jukema J.W. (2015). Ezetimibe Added to Statin Therapy after Acute Coronary Syndromes. N. Engl. J. Med..

[25] Giugliano R.P., Pedersen T.R., Saver J.L., Sever P.S., Keech A.C., Bohula E.A., Murphy S.A., Wasserman S.M., Honarpour N., Wang H. (2020). Stroke Prevention with the PCSK9 (Proprotein Convertase Subtilisin-Kexin Type 9) Inhibitor Evolocumab Added to Statin in High-Risk Patients with Stable Atherosclerosis. Stroke.

[26] Jukema J.W., Zijlstra L.E., Bhatt D.L., Bittner V.A., Diaz R., Drexel H., Goodman S.G., Kim Y.U., Pordy R., Reiner Ž. (2019). Effect of Alirocumab on Stroke in ODYSSEY OUTCOMES. Circulation.

[27] Yokoyama M., Origasa H., Matsuzaki M., Matsuzawa Y., Saito Y., Ishikawa Y., Oikawa S., Sasaki J., Hishida H., Itakura H. (2007). Effects of eicosapentaenoic acid on major coronary events in hypercholesterolaemic patients (JELIS): A randomised open-label, blinded endpoint analysis. Lancet.

[28] Tanaka K., Ishikawa Y., Yokoyama M., Origasa H., Matsuzaki M., Saito Y., Matsuzawa Y., Sasaki J., Oikawa S., Hishida H. (2008). Reduction in the recurrence of stroke by eicosapentaenoic acid for hypercholesterolemic patients: Subanalysis of the JELIS trial. Stroke.

[29] Bhatt D.L., Steg P.G., Miller M., Brinton E.A., Jacobson T.A., Ketchum S.B., Doyle R.T., Juliano R.A., Jiao L., Granowitz C. (2019). Cardiovascular Risk Reduction with Icosapent Ethyl for Hypertriglyceridemia. N. Engl. J. Med..

[30] Ginsberg H.N., Elam M.B., Lovato L.C., Crouse J.R., Leiter L.A., Linz P., Friedewald W.T., Buse J.B., Gerstein H.C., ACCORD Study Group (2010). Effects of combination lipid therapy in type 2 diabetes mellitus. N. Engl. J. Med..

